# Microwell Scaffolds for the Extrahepatic Transplantation of Islets of Langerhans

**DOI:** 10.1371/journal.pone.0064772

**Published:** 2013-05-30

**Authors:** Mijke Buitinga, Roman Truckenmüller, Marten A. Engelse, Lorenzo Moroni, Hetty W. M. Ten Hoopen, Clemens A. van Blitterswijk, Eelco JP. de Koning, Aart A. van Apeldoorn, Marcel Karperien

**Affiliations:** 1 Department of Developmental BioEngineering, MIRA Institute for Biomedical Technology and Technical Medicine, University of Twente, Enschede, The Netherlands; 2 Department of Tissue Regeneration, MIRA Institute for Biomedical Technology and Technical Medicine, University of Twente, Enschede, The Netherlands; 3 Department of Nephrology, Leiden University Medical Centre, Leiden, The Netherlands; 4 Department of BioMedical Chemisty, MIRA Institute for Biomedical Technology and Technical Medicine, University of Twente, Enschede, The Netherlands; 5 Department of Endocrinology, Leiden University Medical Centre, Leiden, The Netherlands; 6 Hubrecht Institute–Royal Netherlands Academy of Arts and Sciences (KNAW) and University Medical Center Utrecht, Utrecht, The Netherlands; Institute for Frontier Medical Sciences, Kyoto University, Japan

## Abstract

Allogeneic islet transplantation into the liver has the potential to restore normoglycemia in patients with type 1 diabetes. However, the suboptimal microenvironment for islets in the liver is likely to be involved in the progressive islet dysfunction that is often observed post-transplantation. This study validates a novel microwell scaffold platform to be used for the extrahepatic transplantation of islet of Langerhans. Scaffolds were fabricated from either a thin polymer film or an electrospun mesh of poly(ethylene oxide terephthalate)-poly(butylene terephthalate) (PEOT/PBT) block copolymer (composition: 4000PEOT30PBT70) and were imprinted with microwells, ∼400 µm in diameter and ∼350 µm in depth. The water contact angle and water uptake were 39±2° and 52.1±4.0 wt%, respectively. The glucose flux through electrospun scaffolds was three times higher than for thin film scaffolds, indicating enhanced nutrient diffusion. Human islets cultured in microwell scaffolds for seven days showed insulin release and insulin content comparable to those of free-floating control islets. Islet morphology and insulin and glucagon expression were maintained during culture in the microwell scaffolds. Our results indicate that the microwell scaffold platform prevents islet aggregation by confinement of individual islets in separate microwells, preserves the islet’s native rounded morphology, and provides a protective environment without impairing islet functionality, making it a promising platform for use in extrahepatic islet transplantation.

## Introduction

Type 1 diabetes is characterized by the autoimmune-mediated destruction of insulin producing β-cells, resulting in absolute insulin deficiency. It is estimated that almost 100,000 children under 15 years of age develop this type of diabetes annually worldwide [1]. Although intensive glucose monitoring combined with exogenous insulin administration can effectively control blood glucose levels, long-term micro- and macrovascular complications, such as nephropathy, retinopathy, neuropathy, and accelerated atherosclerosis, affect many patients [2].

During the last decade, allogeneic islet transplantation in the liver via the infusion of islets into the portal vein has been explored as a potential therapy for patients with type 1 diabetes. Although clinical results for islet transplantation are promising, especially with regard to reducing or eliminating hypoglycemic episodes [3]–[5], widespread application is hindered by the necessity of immunosuppressive agents and by a lack of donor organs. Typically, pancreata from at least two donors are required to achieve normoglycemia in a single patient and insulin independence lasts for only a few years, due to progressive islet loss in the post-transplantation period [3], [4], [6]. It has been estimated that the β-cell volume in islet recipients is only 20–40% that of a healthy person, even when islets are obtained from two to four donors [7]. The islet loss is likely related to the consequences of their injection directly into the portal vein, where they are exposed to several stress factors such as high levels of immunosuppressive drugs, the instant blood-mediated inflammatory reaction (IBMIR), hyperglycemia, and low oxygen tension [7], [8].

These significant disadvantages surrounding intraportal islet transplantation have stimulated the search for extrahepatic, extravascular transplantation sites [8], [9], such as the omental pouch [9], muscle fibers [10], and bioartificial transplantation sites using biomaterials. The advantage of the latter is that the microenvironment can be tailored in order to provide optimal spatial and functional support for the islets, which could ultimately lead to enhanced survival. It has been shown that the efficacy of islet transplantation into adipose tissue can be improved using polymer scaffolds [11], [12].Furthermore, polymer scaffolds immobilize the islets permitting easy transplantation, monitoring of the islet graft after transplantation, and explantation in the case of complications or graft failure. Various scaffold designs, from microporous scaffolds [11]–[18] to hydrogel-based scaffolds [19]–[21], have been assessed both *in vitro* [16], [20] and *in vivo* [11]–[15], [17]–[19], [21] for extrahepatic islet transplantation. However, there remain limitations regarding suitability due to pore geometry and interconnectivity, diffusion rate, stability, and islet fusion.

An interesting class of biocompatible biomaterials for islet transplantation are the poly(ethylene oxide terephthalate) and poly(butylenes terephthalate) (PEOT/PBT) block copolymers. The advantage of these copolymers is that their physical properties and degradation behavior can be tailored by changing the copolymer composition [22]–[27]. Different compositions of these copolymers have been used for *in vivo* skin, bone, and cartilage regeneration and are clinically used as cement stoppers, bone fillers, [28] and as dermal substitutes [29].

The aim of this study is to develop a novel non cell-adhesive, PEOT/PBT microwell scaffold platform to be used for extrahepatic islet transplantation. The advantages of the proposed microwell platform over previously mentioned scaffold designs are that it (1) prevents islet attachment, spreading, and aggregation by the confinement of individual islets in separate microwells preserving the rounded islet morphology; (2) is mechanically stable to protect against physical stresses; and (3) has an open structure permitting fast vascular ingrowth. Polymer thin films and porous meshes, prepared by solvent casting and electrospinning, respectively, were used to fabricate microwell scaffolds by micro back molding. Scaffolds were characterized for their wettability, water uptake, nutrient diffusion, and cytotoxicity. Subsequent *in vitro* experiments demonstrated that human islets cultured in the microwell scaffolds retained their native morphology and their insulin secretion was comparable to that of free-floating control islets indicating that this novel scaffold platform does not hamper islet functionality. Our data therefore indicate that the PEOT/PBT microwell scaffold is a potential carrier for extrahepatic islet transplantation.

## Methods

### PEOT/PBT Thin Film Fabrication

The chemical composition of PEOT/PBT polymer is indicated as aPEOTbPBTc, in which ‘a’ is the molecular weight of the starting poly(ethylene glycol) PEG segments used in the polymerization process, while ‘b’ and ‘c’ refer to the weight percentage of PEOT and PBT blocks, respectively. The composition 4000PEOT30PBT70 was selected for its slow degradation rate and its limited cell-adhesive properties. The thin film microwell scaffolds were prepared from ∼50 µm thick 4000PEOT30PBT70 block copolymer films (IsoTis Integra Orhobiologics S.A., Irvine, USA). These polymer films were fabricated by solvent casting. A 10% (w/w) polymer solution was prepared in 20% (w/w) 1,1,1,3,3,3-hexafluoro-2-isopropanol (Biosolve, Valkenswaard, The Netherlands) and 80% (w/w) chloroform (Merck, Darmstadt, Germany) and casted on a silicon wafer at ambient temperature (23±3°C). The films were placed under nitrogen-flow for 12 hours to form dense films, incubated in ethanol overnight to remove solvent residue, and dried in a vacuum oven (Heraeus, Hanau, Germany) at 30°C for 3 days.

### PEOT/PBT Electrospun Mesh Fabrication

Electrospun microwell scaffolds were fabricated from 150 µm thick electrospun 4000PEOT30PBT70 block copolymer meshes. The meshes were fabricated from a 10% (w/w) polymer solution in 20% (w/w) 1,1,1,3,3,3-hexafluoro-2-isopropanol and 80% (w/w) chloroform using an electrospinning apparatus as previously described [30]. Electrospinning was performed at 12 kV constant voltage, with a 1.2 mm diameter needle, a 10 cm distance between the syringe and the collector, and a flow rate of 2 ml/h. Electrospun meshes were dried in a vacuum oven (Heraeus, Hanau, Germany) at 30°C for 3 days.

### Microwell Scaffold Fabrication

Microwell scaffolds were fabricated from the PEOT/PBT thin films and electrospun meshes by micro back molding [31], a variant of microthermoforming, which involves shaping a heated polymer film by three-dimensional stretching into a negative mold using a backing material (Figure S1) [32]–[35]. The negative mold was produced by Lightmotif BV (Enschede, The Netherlands) out of a 350 µm thick stainless steel (AISI 304) foil. Approximately 500 circular holes with a diameter of 400 µm were ablated by a titanium-sapphire laser (800 nm wavelength, circular polarized, 50 kHz pulse repetition rate, 5 µJ energy, and 200 fs pulse duration). A 25 µm laser spot was achieved by a 100 mm focal length lens. The laser bundle was scanned over the foil by a two-mirror galvanometer (Scanlab Scangine 14). After machining, the mold was cleaned in an ultrasonic bath. Molding temperature and pressure for thin film scaffolds were 85°C and 25 kN, respectively and for the electrospun microwell scaffolds they were 17°C and 3 kN, respectively. Scaffold geometry and architecture were characterized by scanning electron microscopy (SEM) on a Philips XL 30 ESEM-FEG. Scaffolds were gold sputter coated (Cressington, UK) prior to SEM analysis. The dimensions of the microwells (n = 10) were measured in three different samples per type of scaffold using ImageJ software (http://rsb.info.nih.gov/ij/).

### PEOT/PBT Wettability and Water Uptake

To assess the effect of micro back molding on polymer wettability and water uptake, 4000PEOT30PBT70 block copolymer films were heat-treated (85°C) to simulate the scaffold fabrication cycle. The wettability was determined by static water contact angle measurements using the captive bubble method before and after heat-treatment. Measurements were performed using a video-based optical contact angle meter OCA 15 (DataPhysics Instruments GmbH, Filderstadt, Germany). The polymer films were mounted onto Scaffdex CellCrown inserts (Tampere, Finland) and incubated for three days in ultrapure Milli-Q water at 37°C. For measurements, the samples were placed with the flat polymer surface downward in an optical cuvette filled with ultrapure Milli-Q water. Water contact angle was determined by applying an air bubble (∼10 µL) on the film using an electronically regulated Hamilton syringe containing a curved needle. The contact angle was calculated using SCA20 software (DataPhysics Instruments GmbH, Filderstadt, Germany). The equilibrium water-uptake in ultrapure Milli-Q water was defined as the percentage weight gain of the polymer film after incubation at 37°C for 7 days. The swollen weight was measured after blotting the films with filter paper to remove surface water using a high-precision balance with an accuracy of ±0.01 mg (Sartorius BP210D, Göttingen, Germany).

### PEOT/PBT Cytotoxicity

Heat-treated (85°C) 4000PEOT30PBT70 block copolymer films were tested for cytotoxicity using a minimum essential medium (MEM) extract test (adapted from ISO 10993/EN 30993 standard). Small pieces of the polymer films (total surface of 36 cm^2^) were sterilized in 70% (v/v) ethanol overnight and dried for at least 12 hours. For extraction, the films were incubated in medium (α-MEM (Invitrogen, Carlsbad, USA) supplemented with 2 mM l-glutamine, 1 mM sodium pyruvate, 10% FBS, 100 U/ml penicillin and 10 µg/ml streptomycin) for 72 hours in a shaking water bath (37°C, 60 rpm). Natural rubber (Gelria Packing BV, Enschede, The Netherlands), polylactic acid films (PLA; Plastic Suppliers/Sidaplax, EarthFirst Packaging Film, Gentbrugge, Belgium), and polypropylene films (Walothen O 25 E, Germany) were extracted identically and used as a positive control, a reference, and a negative control for cytotoxicity, respectively. The extracts were added to a subconfluent monolayer of MC3T3-E1 cells (mouse pre-osteoblasts) and cultured for 72 hours. After 24, 48 and 72 hours the cells were evaluated under light microscopy and scored in a single-blinded manner three times for confluency, degree of floating cells, and change of cell morphology related to the negative control. After 72 hours, the cells were trypsinized and counted (Beckman Coulter, Miami, USA). Based on these results, the cytotoxic response index was scored according to ISO 10993/EN 30993 standard.

### Nutrient Diffusion through Microwell Scaffolds

As an indicator for nutrient transport through thin film and electrospun scaffolds, glucose diffusion was assessed as previously described [36]. Briefly, the setup consisted of two double-walled compartments: a donor compartment filled with RPMI-1640 (Gibco) containing 11.1 mM d-glucose, and an acceptor compartment containing RPMI-1640 without d-glucose (both compartments were supplemented with 100 U/ml penicillin and 10 µg/ml streptomycin). The scaffolds were pre-wetted for several hours with an ethanol to medium (without d-glucose) gradient and placed between the two compartments which were maintained at 37°C with circulating water. At the indicated time intervals, 80 µl samples were obtained from both compartments and analyzed for glucose concentration using a Vitros DT60 II chemistry system (Ortho-Clinical Diagnostics, Raritan, USA). The glucose flux, the amount of glucose that passes through a certain surface in time, was calculated using the equation:
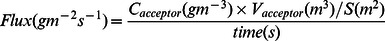
(1)where C_acceptor_ is the glucose concentration in the acceptor compartment, V_acceptor_ is the volume of the acceptor compartment, and S, the surface area of the construct.

### Islet Isolation and Seeding in Microwell Scaffolds

Human islets of Langerhans from pancreata of organ donors (n = 4) were obtained from the Human Islet Isolation Laboratory at the Leiden University Medical Center (Leiden, The Netherlands), which has permission from the Dutch government to isolate human islets for clinical purposes. Human islets of four different donor organs that were not eligible for clinical transplantation were used in these experiments, in accordance with Dutch law and institutional requirements.

Microwell scaffolds were sterilized in 70% (v/v) ethanol overnight and washed in PBS. Before islet seeding, scaffolds were centrifuged in islet culture medium (CMRL-1066 (Mediatech, Cellgro, Herndon, USA) containing 5.5 mM d-glucose, and supplemented with 0.02 mg/ml ciproxin, 0.05 mg/ml gentamicin, 2 mM l-glutamine, 0.25 µg/ml fungizone, 10 mM HEPES, 1.2 mg/ml nicotinamide and 10% (v/v) human serum) at 3500 rpm for 5 minutes to remove air bubbles and were mounted between two Teflon rings. Islets were handpicked and seeded drop-wise onto scaffolds. After 5 minutes of incubation, 8 ml of culture medium was added. Medium was changed every other day. As a reference, islets were also cultured free-floating in ultra-low attachment petri-dishes (Corning, New York, USA).

### Islet Morphology and Function in Microwell Scaffolds

To assess morphology and viability, human islets were cultured in thin film and electrospun microwell scaffolds for 7 days. To assess islet morphology, samples were fixed in 4% (w/v) paraformaldehyde for 2 hours at ambient temperature (23±3°C), permeabilized with 0.1% Triton-X for 4 minutes and stained with alexa-488-phalloidin for 30 minutes in the dark to visualize actin in islet cells. Samples were imaged using a Zeiss LSM510 confocal MicroscopeAdditionally, islet morphology was assessed using SEM. As a reference for islet attachment, islets were cultured for 7 days on tissue culture plastic (NUNC ).Prior to SEM analysis, samples were fixed in 4% (w/v) paraformaldehyde for 2 hours at ambient temperature (23±3°C), dehydrated in graded ethanol series, critical-point dried, and gold sputter coated.

To assess islet function, two measures of islet response to glucose were made: insulin secretion and total insulin content were assessed at day 1 and day 7. Scaffolds containing islets or free-floating islets were transferred to ultra-low attachment 24-well plates (Corning, New York, USA). Each well contained approximately 25 islets. The islets were incubated in pre-warmed KRBH buffer (115 mM NaCl, 5 mM KCl, 24 mM NaHCO_3_ and 2.2 mM CaCl_2_, pH 7.4), supplemented with 20 mM HEPES, 2 mg/ml human serum albumin and 1.7 mM d-glucose for a 90 minutes at 37°C and 5% CO_2_. The buffer was replaced by KRBH buffer containing either 1.7 mM or 16.7 mM glucose and the islets were incubated for 60 minutes. Experiments were performed in triplicate for each condition. The supernatant was removed and frozen for future analysis. Islets were harvested, washed in PBS and disrupted by sonication in distilled water (Braun, Melsungen, Germany). Samples from the homogenate were extracted overnight at 4°C in acidic ethanol (0.18 M HCl in 95% (v/v) ethanol). The supernatants and islet homogenate were assayed for insulin using an ELISA immunoassay (Mercodia, Uppsala, Sweden) according to the manufacturer’s instructions. For normalization, total DNA content was quantified using a Quant-iT PicoGreen dsDNA kit (Molecular Probes, Eugene, USA) according to the manufacturer’s instructions.

### Histological Analysis

To identify α and β cells and assess morphology, histology was performed. Islets cultured in either microwell scaffolds or under free-floating conditions were washed in PBS and fixed in 4% (w/v) paraformaldehyde for 2 hours at ambient temperature (23±3°C). Subsequently, the samples were embedded in 2% (w/v) agarose and prepared for paraffin embedding. The samples were sectioned at 4 µm, deparaffinized in buthylene, and rehydrated through graded ethanol series to distilled water. Sections were blocked with an avidin-biotin blocking kit (Vector Laboratories, Burlingame, USA)) and normal donkey serum (1∶50 (in PBS containing 1% lamb serum), Jackson ImmunoResearch, West Grove, USA, 1 hour at room temperature). Primary and secondary antibodies were diluted in PBS containing 1% lamb serum. The following primary antibodies, dilutions, and incubation times were used: guinea pig anti-insulin (1∶200, Linco Research, St Charles, USA, 1.5 hours at room temperature) and rabbit anti-glucagon (1∶100, Vector Laboratories, Burlingame, USA, overnight at 4°C). The secondary antibodies were donkey anti-guinea pig-TRITC (1∶400, Jackson ImmunoResearch, West Grove, USA, 1 hour at room temperature) and biotinylated donkey anti-rabbit (1∶200, Jackson ImmunoResearch, West Grove, USA, 1 hour at room temperature) followed by streptavidin-Alexa Fluor488 (1∶200 Jackson ImmunoResearch, West Grove, USA, 1 hour at room temperature). Sections were mounted with mounting medium containing DAPI to stain the nuclei (VectaShield, Vector Laboratories, Burlingame, USA) prior to imaging on a confocal microscope (Carl Zeiss, Sliedrecht, the Netherlands).

### Statistical Analysis

Results are presented as the mean ± standard deviation. Statistical analyses were performed using an unpaired, two-tailed, Student’s t-test, or analysis of variance (ANOVA) with a Bonferroni post-test using SPSS statistics software (Chicago, USA). Statistical significance was considered at *p*<0.05.

## Results

### Polymer Film Characterization and Cytotoxicity

**Figure 1:**
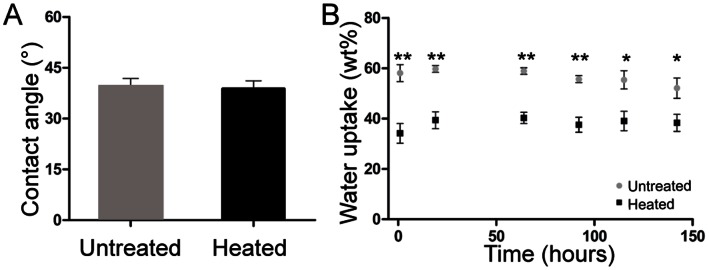
PEOT/PBT wettability and water uptake. (A) Contact angle measurements of non heat-treated and heat-treated 4000PEOT30PBT70 block copolymer films using the captive bubble method. Data represents mean ± SD of 20 measurements (*N* = 3 per condition). (B) Water uptake of non-treated and heat-treated 4000PEOT30PBT70 block copolymer films. Data represents mean ± SD, (*N* = 3 per condition), *p<0.05, **p<0.01.

**Figure 2:**
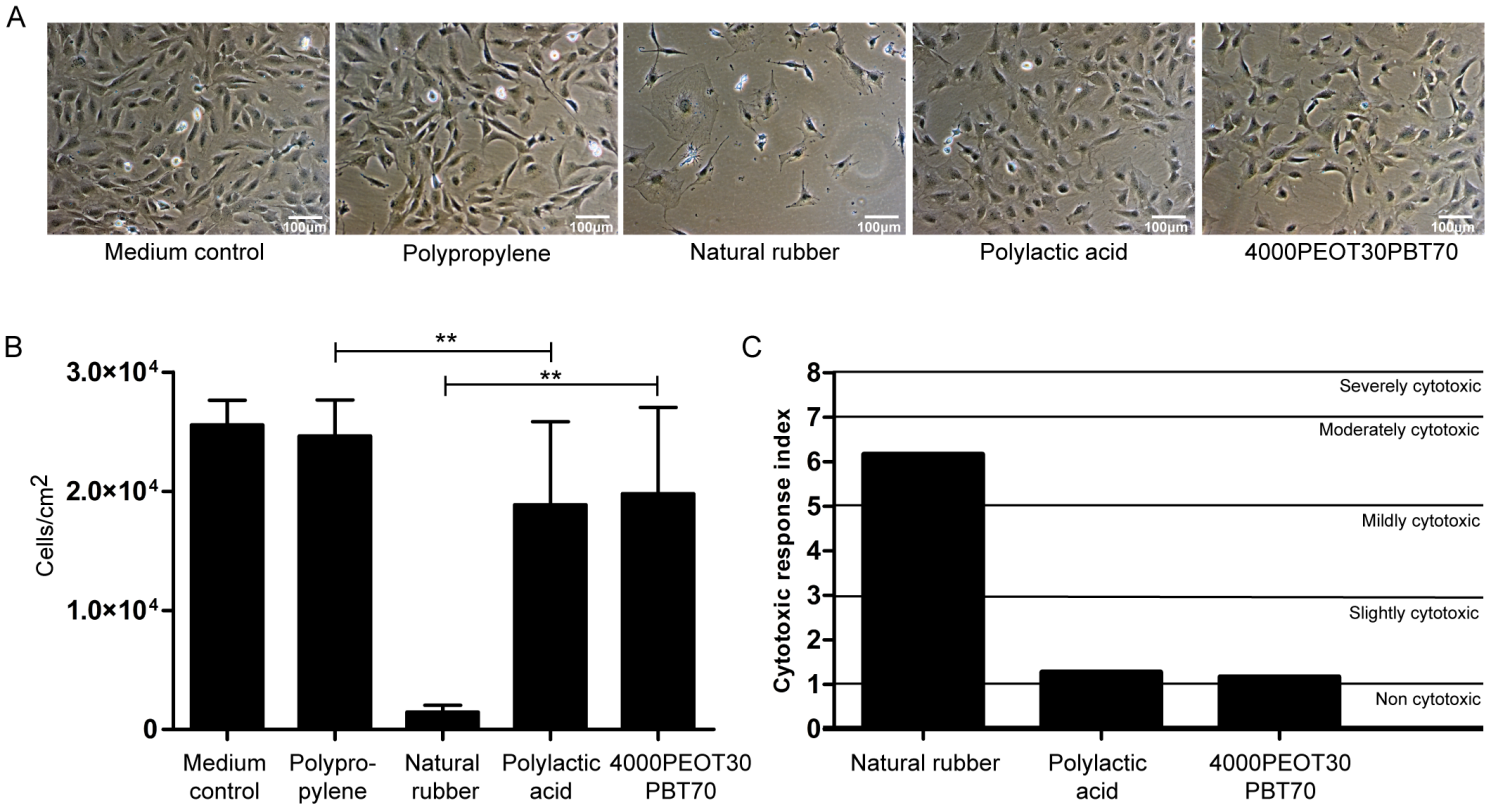
PEOT/PBT cytotoxicity. Cytotoxicity test after 72 hours of incubation. Growth and morphology of MC3T3E-1 cells were analyzed in the presence of extracts of polypropylene films (negative control), natural rubber (positive control), polylactic acid and heated 4000PEOT30PBT70 films. (A) Light microscopy images of MC3T3E-1 cells cultured in standard and in the presence of the different material extracts, (B) Total amount of cells after 72 hours of incubation. Data represent mean ± SD, (n = 6), **p<0.01, (C) The cytotoxic response index based on growth and morphological changes, scored in a single-blinded manner.

We first determined the effects of our processing conditions on the 4000PEOT30PBT70 block copolymer. Water uptake and contact angle of the unprocessed polymer was 52.1±4.0% (w/w) and 39±2°, respectively. After heating the polymer to 85°C, the temperature used for scaffold fabrication, water uptake was significantly decreased to 38.3±3.4% (w/w), whereas no change in water contact angle was observed (Figure 1). We then sought to ensure the processing conditions did not affect the biocompatibility. To evaluate the cytotoxicity of heat-treated 4000PEOT30PBT70 block copolymer films, extracts of these materials were tested for their effects on growth and morphology of MC3T3-E1 cells. In contrast to exposure to extracts of natural rubber, which served as the positive control for cytotoxicity, nearly confluent monolayers were observed after exposure to extracts of polypropylene (negative control), PLA, and 4000PEOT30PBT70 films (Figure 2 A–B). No significant effect on cell morphology was observed for cells cultured in 4000PEOT30PBT70 extracts compared to PLA or polypropylene, indicating the polymer is biocompatible.

### Microwell Scaffold Morphology

**Figure 3:**
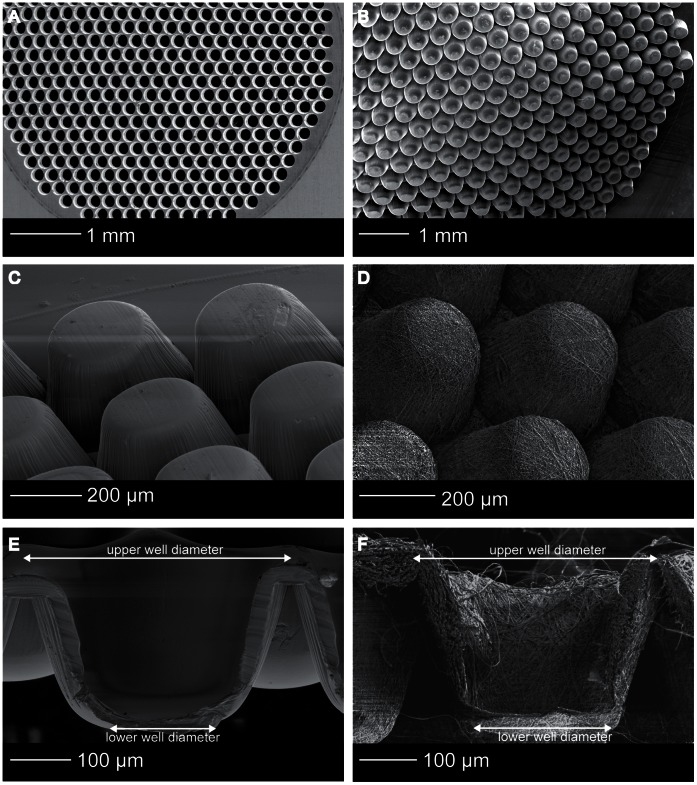
Microwell scaffold morphology. SEM images of (A) the stainless steel mould, (B) the scaffold area, (C) thin film microwell scaffold, (D) electrospun microwell scaffold, (E) cross-section of a thin film microwell showing the tapered shape and the smooth polymer surface, (F) cross-section of a electrospun microwell showing the fibrous network.

SEM analysis demonstrated homogeneously shaped microwells throughout the entire scaffold area (Figure 3 B), resembling the tapered shape of the mold, (Figure 3 A) and indicating successful demolding. The microwells were stable upon handling, due to the sufficiently high ratios of the thicknesses of the walls to the dimensions of the volumes enclosed by the walls. Per scaffold type, the upper and lower well diameters (Figure 3 E–F) of 10 microwells in three different samples were measured using ImageJ software (http://rsb.info.nih.gov/ij/). The upper well diameter of the thin film scaffolds was 377±5 µm and the lower well diameter 251±9 µm. For the electrospun microwells the upper diameter was 355±20 µm and the lower diameter was 217±36 µm. The fiber-diameter of the electrospun meshes was 1.71±0.42 µm. The height of the scaffold corresponded to the height of the mold (350 µm) (Figure 3 C–F).

### Nutrient Diffusion through Microwell Scaffolds

**Figure 4:**
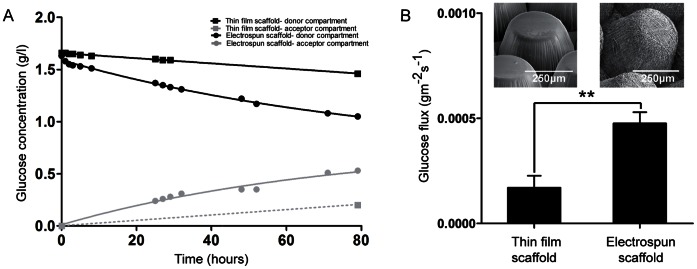
Glucose diffusion through microwell scaffolds. (A)Typical glucose diffusion graphs for thin film and electrospun microwell scaffolds. For the thin film microwell scaffold, the concentration of glucose in the acceptor compartment is represented as a dashed line, because only the last time-point was within analytical range of the Vitros DT60 II chemistry system. (B) Maximum glucose flux through dense and electrospun microwell scaffolds. Data represents mean ± SD (n = 3), **p<0.01.

As glucose is an important nutrient for islets, the diffusion of glucose through thin film and electrospun microwell scaffolds was assessed. Figure 4 A shows typical results of glucose diffusion in time through a thin film and an electrospun scaffold. No adsorption of glucose to the scaffolds was observed, since the difference between the initial concentration of glucose in the donor compartment and the concentration in the donor compartment in time equals the glucose concentration in the acceptor compartment. Due to the water uptake of this polymer composition, some glucose diffusion was observed through thin film microwell scaffolds (Figure 4 A), but the maximum glucose flux was significantly higher for the electrospun scaffolds: 4.76±0.53E10^−4^ gm^−2^s^−1^ compared to 1.70±0.58E10^−4^ gm^−2^s^−1^ for the thin film scaffolds (*p*<0.01) (figure 4 B).

### Islet Morphology in Microwell Scaffolds

**Figure 5:**
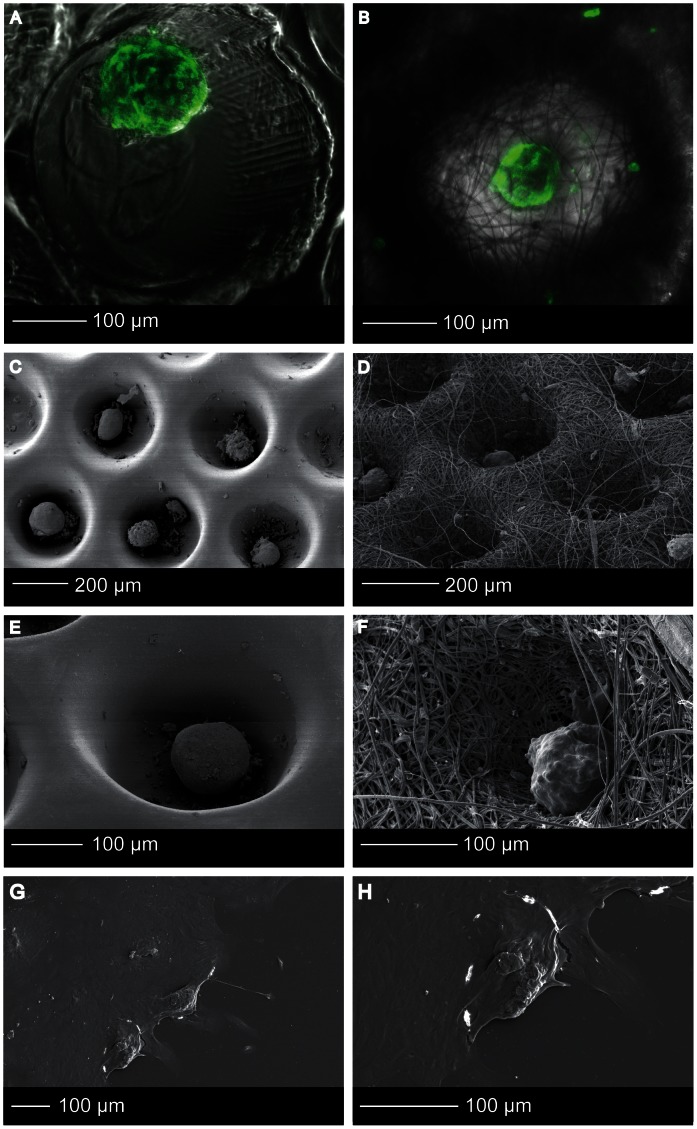
Islet morphology. (A, B) Confocal fluorescence microscopy images of Phalloidin-TRITC stained human islets that were cultured in thin film (A) and electrospun (B) microwell scaffolds. (C–H) High resolution SEM images of islets in the thin film (C,E) and electrospun (D,FD) microwell scaffolds, and tissue culture plastic (G,H).

Islets were seeded into the scaffolds by sedimentation. It is known that islets aggregate when they are cultured at high density. In rare cases, multiple islets were observed in one microwell. After prolonged culture, these islets start to fuse, but the final size of the fused islets remained within the dimensions of the microwell (∼350 µm), comparable to the upper size limit of human islets [37]. After 7 days of culture, the cells did not spread out and islets maintained their rounded shape (Figure 5 A–F) in both thin film and electrospun mesh microwell scaffolds. This in contrast to islets that were cultured on adherent tissue culture plastic used as an islet adhesion control (Figure 5 G–H). Some attached cells could be observed at the surface of the scaffold, but the degree of cell attachment was limited.

### Islet Response to Glucose Challenge

**Figure 6:**
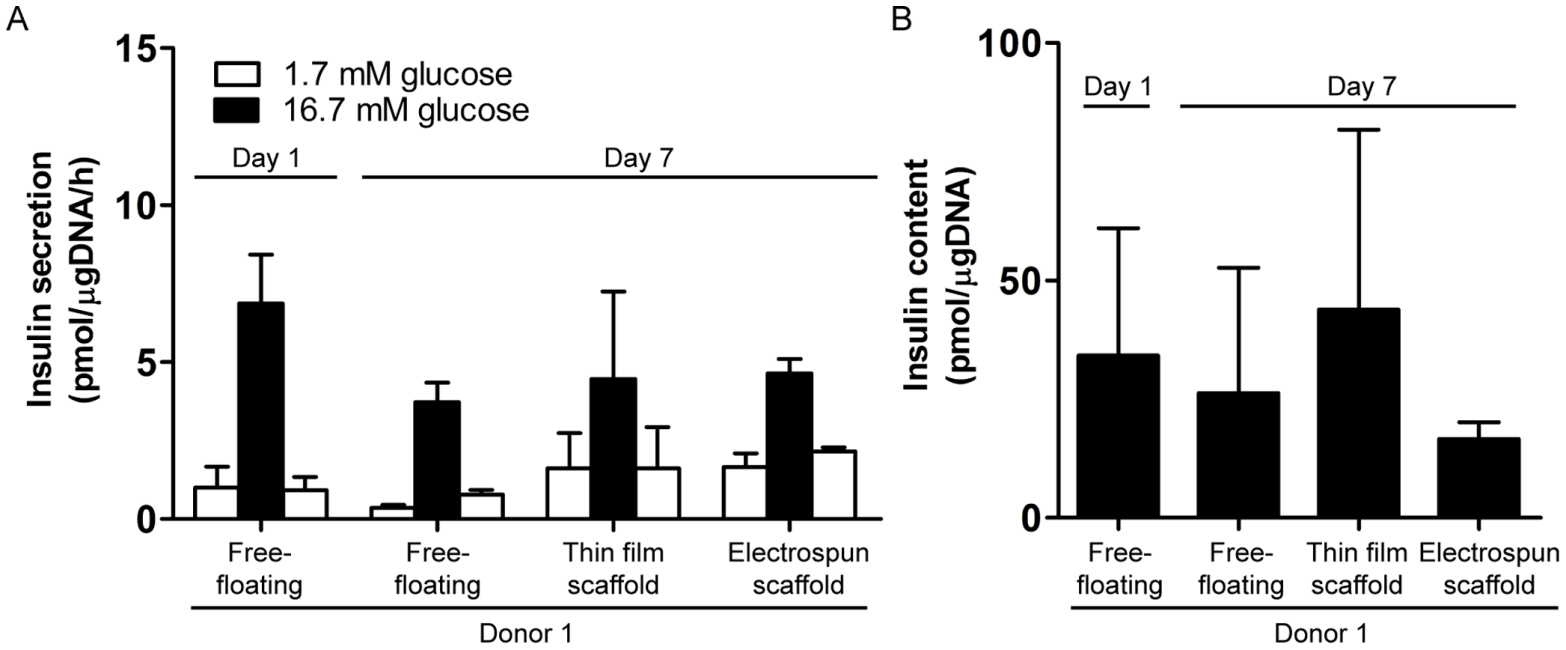
Islet function in microwell scaffolds. (A) Insulin secretory response to glucose stimulation of human islets cultured for 7 days in thin film and electrospun microwell scaffolds. Free-floating islets served as a control. Data represent mean ± SD (n = 3 per condition). (B) Insulin content of human islets cultured in thin film and electrospun microwell scaffold or under free-floating conditions. Data represent mean ± SD (n = 3 per condition).

**Figure 7:**
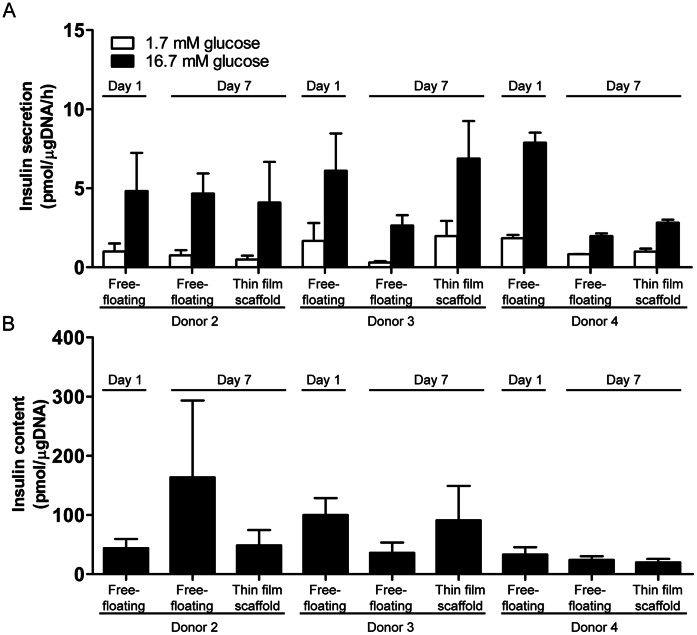
Islet function of multiple donors in thin film microwell scaffolds. (A) Insulin secretory response and, (B) insulin content of islets cultured in thin film scaffolds or under free-floating conditions of three different human donors. Data represent mean ± SD (n = 3 per condition).

To assess whether the microwell scaffolds affect islets functionality, human islets were cultured for 7 days in both thin film and electrospun microwell scaffolds and subjected to a glucose-stimulated insulin secretion (GSIS) test. This test involves exposing islets to low (1.7 mM), high (16.7 mM), and then low glucose concentration and measuring insulin secretion by ELISA. After 7 days of culture, the increase in insulin release upon stimulation, compared to basal insulin release levels, did not significantly differ between islets seeded in either scaffold design (3.9±2.9 and 3.6±1.6 pmol/µgDNA/h for thin film and electrospun microwell scaffolds, respectively) and free-floating control islets (3.6±0.7 pmol/µgDNA/h, *p*>0.05). Following high glucose stimulation, insulin release levels returned to basal levels when incubated in low glucose buffer, indicating normal responsiveness (Figure 6 A). Total insulin content was also measured and did not differ between islets in the microwells or free-floating control islets (Figure 6 B). With no significant differences in insulin secretion between islets cultured in either the thin film or the electrospun mesh microwells, we sought to further demonstrate that donor variability was not influencing our results. We therefore compared insulin secretion and total insulin content in islets derived from three donors either in thin film microwells and free-floating islets and found no statistically significant differences (Figure 7 A–B) indicating that the microwell scaffold platform does not hamper islet functionality in terms of islet’s glucose-induced insulin secretion.

### Histological Analysis

**Figure 8:**
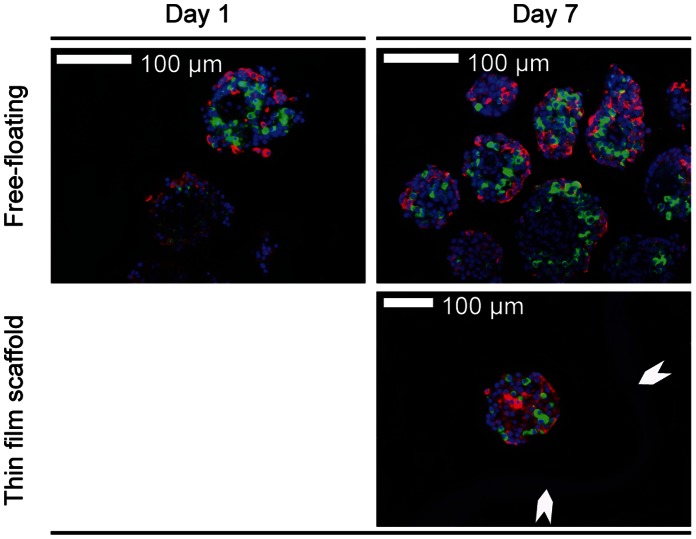
Histology of free-floating control islets and of islets cultured in thin film microwell scaffolds. Insulin (red) and glucagon (green) positive cells can be observed throughout the islets. The microwell scaffold is indicated by the white arrows. All data were obtained after 7 days of culturing.

Having found that both the thin film and the electrospun microwell scaffolds supported islet insulin response equally, we performed histology of islets seeded in the thin film scaffold and compared this to free-floating islets. Islet morphology was unaffected by culture in the thin film microwell scaffolds after 7 days of culture (Figure 8). In both groups, glucagon and insulin expressing cells were typically present throughout the islets, further indicating that the microwell scaffolds did not negatively affect islet morphology nor function.

## Discussion

Biomaterial scaffolds have recently been proposed as a carrier for islets of Langerhans to improve long-term islet survival and function *in vivo*. They avoid triggering the instant blood-mediated inflammatory reaction (IBMIR) and may provide a better microenvironment for islet transplantation than transplantation in the suboptimal microenvironment of the liver vasculature [38]. This study represents the first use of a microwell scaffold platform designed for future islet of Langerhans transplantation. This microwell design permitted easy entrapment of individual islets through sedimentation and the dimensions covered the full size range of the islets [37]. In other scaffolds, filling materials such as matrigel [12] and type I collagen gels [16] were used to prevent islet loss during seeding but the microwell scaffold design presented here entraps the islets without the need for additional filling materials even after multiple media changes. The open structure of the microwell scaffold may be advantageous for nutrient diffusion and infiltration of host cells, such as endothelial cells, which are important for islet survival, function and engraftment. This addresses a common problem post-transplantation, where nutrients can only be supplied to the islets through diffusion until the islets are revascularized, which can take up to 14 days. Even then, the vascular density and oxygen tension in these transplanted islets are reduced compared to native islets [39]. SEM analysis further revealed that the natural rounded morphology of islets was preserved in the scaffolds, regardless of whether they were seeded in thin films or electrospun meshes.

For this study, a PEOT/PBT block copolymer was used, since its material properties, such as wettability [24], swelling [23], [25], [27], protein adsorption [26], degradation rate [27], and mechanical properties [27] can be tailored for specific applications by changing the copolymer composition [27]. The composition 4000PEOT30PBT70 was selected for its slow degradation rate, allowing long-term monitoring after transplantation, and its limited cell-adhesive properties [24] due to the high molecular weight of the initial PEG segments [40]. Importantly, our fabrication process did not affect the polymer film wettability, but the water uptake was significantly diminished. The temperature during scaffold fabrication (85°C) was above the PEOT-domain melting temperature (32–47°C [27]), but below the melting temperature of the PBT-domain (173–223°C [27]). Atomic force microscopy showed the distribution of the two different blocks was more organized after heating, indicative of greater phase separation (Figure S2), potentially explaining the diminished water uptake.

Considering that pH is important for islet function and survival [41] and that long-term exposure to non-physiological pH has been demonstrated to result in a loss of islets when below 7.0 and loss of the insulin releasing capacity when above 7.4 [41], it is important that the PEOT/PBT copolymer does not affect the local pH upon degradation. Previous *in vitro* and *in vivo* work has demonstrated that non-enzymatic PEOT/PBT degradation, which is primarily through hydrolysis, is unlikely to affect the pH of the local environment. This is a significant advantage over polymers such as poly(glycolic acid) (PGA) [31], [32] and poly(lactic-*co*-glycolic acid) (PLGA) [27]–[29], which are often used in scaffolds for islet transplantation in which acidic degradation products are formed.

The microwell design confined individual islets in separate microwells, overcoming a major disadvantage of previous scaffold designs in which islets were able to fuse. Islet fusion negatively affects native islet structure, and results in islet aggregates which suffer from diffusion-limited nutrient deprivation [37]. This results in central core necrosis and impaired islet function. Furthermore, our design preserved the native islet morphology during 7 days of culture. The rounded islet morphology is thought to be crucial for islet function, as a higher ratio between the basal and glucose-stimulated insulin release is obtained when islets are cultured free-floating compared to islets that are attached to the bottom of a petri-dish [42]. Functional tests revealed that the insulin secretory response to glucose stimulation of human islets cultured in the microwell scaffolds was comparable to free-floating control islets. Also the insulin content of scaffold-seeded islets was preserved indicating that there is no change in insulin release and pro-insulin biosynthesis.

The translation of the microwell system to a clinical setting raises some additional challenges; for example, Shapiro *et al.* [5] have shown that a mean of 11,547±1604 islet equivalents (IEQ) per kilo bodyweight is needed to obtain insulin independency in patients. Therefore, to be able to transplant the required islet mass to treat a patient of 75 kilograms using a single microwell, the scaffold diameter would have to increase to 40 cm. Even though the height of the scaffold is only 350 µm, it would not be clinically feasible to transplant a scaffold with such dimensions. Thus, before translation of this work to clinically relevant sized implants, we will study methods to scale up the scaffold platform to accommodate a sufficient amount of islets for human islet transplantation. We calculated that multi-layer stacking of microwell scaffolds into a three-dimensional scaffold will already drastically reduce the construct’s dimensions: stacking up to 15 scaffold layers will result in a three-dimensional construct with a diameter of 10 cm and a height of 5 mm. When stacking multiple scaffold layers, one should consider diminished mass transport of nutrients and hormones. Therefore, our future studies will focus on introducing micro-porosity into the microwell scaffolds, which will benefit the three-dimensional microwell scaffold design two-fold. First, the enhanced nutrient diffusion will improve islet survival immediately after transplantation, while secondly, the micro-pores allow vascular ingrowth into three-dimensional construct to revascularize the islets rapidly.

### Conclusions

This study is the first to report on a microwell scaffold platform as a potential transplantation device for pancreatic islets. Reproducible microwell scaffolds were prepared from PEOT/PBT thin films and electrospun meshes. The morphology of human islets was preserved in the microwell scaffolds and remained stable during 7 days of culture and the islets showed an insulin release and total insulin content comparable to free-floating control islets. Glucagon and insulin immunostaining were also comparable between these groups. Together, these data indicate that the microwell scaffold platform does not hamper islet functionality and is a promising platform for use in extrahepatic islet transplantation.

## Supporting Information

Figure S1 **Schematic representation of scaffold fabrication process using micro back molding.** (1) press, (2) backing material, (3) thin polymer film or electrospun mesh of 4000PEOT30PBT70 block copolymer, (4) mold, (5) formed thin polymer film or electrospun mesh, (6) solidified backing, (7) microthermoformed microwell scaffold.
(TIF)

Figure S2 **Height and corresponding phase contrast images obtained by tapping mode Atomic Force Microscopy (AFM).** (A–C) untreated 4000PEOT30PBT70 block copolymer films; (B–D) heated 4000PEOT30PBT70 block copolymer films. Phase contrast images (C–D) show the PEOT/PBT domains for both samples. Tapping mode imaging was performed on a Multimode with a Nanoscope IV controller (Bruker, Santa Barbara CA, USA) with TESP cantilevers (spring constant is 20–80 N/m, Bruker, Santa Barbara CA, USA) in ambient air with moderate force settings (65% of the free amplitude). Image size is 1×1 µm with 512 pixels.
(TIF)
