# Supplementary figures and images for: Microwell Scaffolds for the Extrahepatic Transplantation of Islets of Langerhans
Source: PLoS One. 2013 May 30;8(5):e64772. doi: 10.1371/journal.pone.0064772 (PMC3667808; doi:10.1371/journal.pone.0064772)

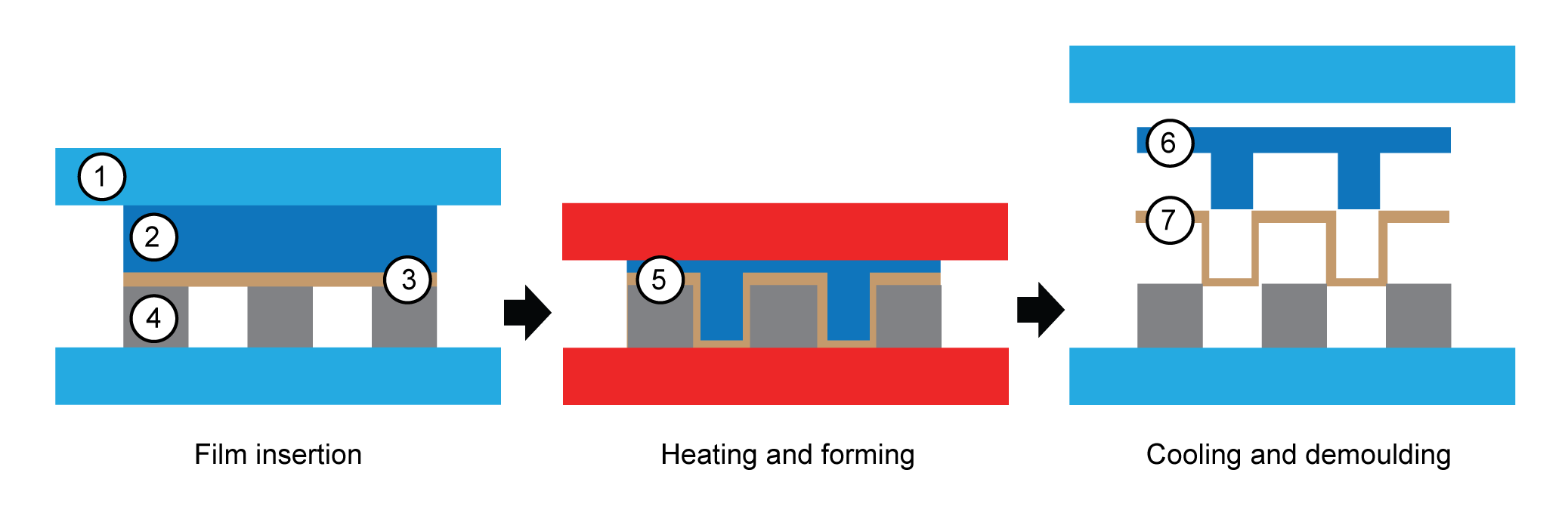

Supplement: Figure S1 — Schematic representation of scaffold fabrication process using micro back molding. (1) press, (2) backing material, (3) thin polymer film or electrospun mesh of 4000PEOT30PBT70 block copolymer, (4) mold, (5) formed thin polymer film or electrospun mesh, (6) solidified backing, (7) microthermoformed microwell scaffold. (TIF) [file pone.0064772.s001.tif]

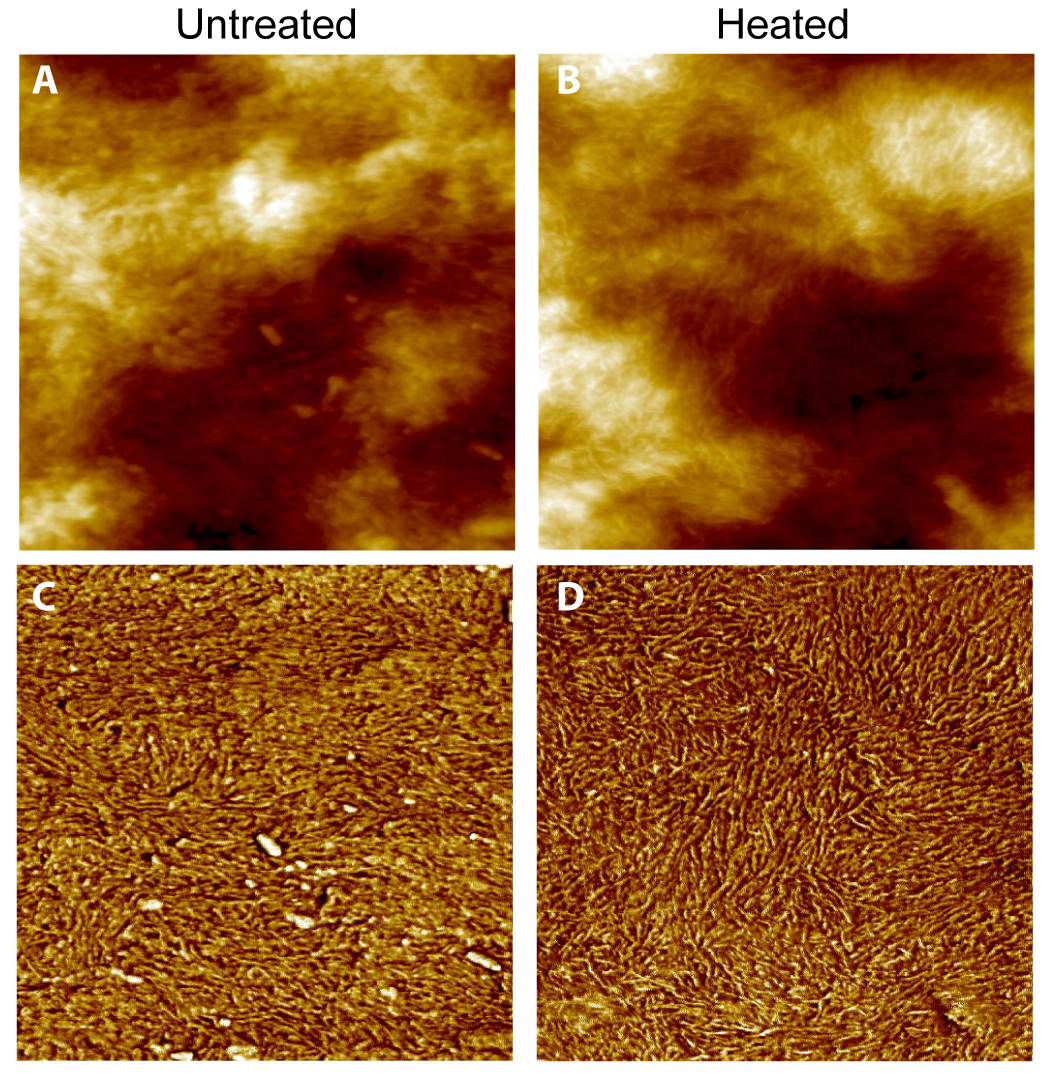

Supplement: Figure S2 — Height and corresponding phase contrast images obtained by tapping mode Atomic Force Microscopy (AFM). (A–C) untreated 4000PEOT30PBT70 block copolymer films; (B–D) heated 4000PEOT30PBT70 block copolymer films. Phase contrast images (C–D) show the PEOT/PBT domains for both samples. Tapping mode imaging was performed on a Multimode with a Nanoscope IV controller (Bruker, Santa Barbara CA, USA) with TESP cantilevers (spring constant is 20–80 N/m, Bruker, Santa Barbara CA, USA) in ambient air with moderate force settings (65% of the free amplitude). Image size is 1×1 µm with 512 pixels. (TIF) [file pone.0064772.s002.tif]
